# Mechanisms of Male Reproductive Sterility Triggered by Dysbiosis of Intestinal Microorganisms

**DOI:** 10.3390/life14060694

**Published:** 2024-05-28

**Authors:** Mingbang Wei, Huaizhi Liu, Yu Wang, Mingyang Sun, Peng Shang

**Affiliations:** 1 College of Animal Science, Tibet Agriculture and Animal Husbandry University, Linzhi 860000, China; bangbangbang962@163.com (M.W.); lhzhyyy@163.com (H.L.); 15237208136@163.com (Y.W.); smy981225@163.com (M.S.); 2 The Provincial and Ministerial Co-Founded Collaborative Innovation Center for R & D in Tibet Characteristic Agricultural and Animal Husbandry Resources, Linzhi 860000, China; 3 Key Laboratory for the Genetic Improvement and Reproduction Technology of the Tibetan Swine, Linzhi 860000, China

**Keywords:** gut microbiota, male reproductive infertility, immune system, endocrine system, gut–brain–reproductive axis

## Abstract

The intestinal microbiota, comprised of bacteria, archaea, and phages, inhabits the gastrointestinal tract of the organism. Male reproductive sterility is currently a prominent topic in medical research. Increasing research suggests that gut microbiota dysbiosis can result in various reproductive health problems. This article specifically investigates the impact of gut microbiota dysbiosis on male reproductive infertility development. Gut microbiota imbalances can disrupt the immune system and immune cell metabolism, affecting testicular growth and sperm production. This dysfunction can compromise the levels of hormones produced and secreted by the endocrine glands, affecting male reproductive health. Furthermore, imbalance of the gut microbiota can disrupt the gut–brain–reproductive axis, resulting in male reproductive infertility. This article explores how the imbalance of the gut microbiota impacts male reproductive infertility through immune regulation, endocrine regulation, and interactions of the gut–brain–reproductive axis, concluding with recommendations for prevention and treatment.

## 1. Introduction

Infertility affects about 10–15% of couples globally, with men of reproductive age accounting for 50% of the determinants [1]. Male infertility can be classified into congenital and acquired causes. Congenital factors primarily involve impaired testicular development during fetal growth in the mother’s womb, leading to congenital testicular deficiency and subsequent male infertility. On the other hand, acquired causes encompass a spectrum of problems, including reproductive system infections, endocrine abnormalities, unhealthy lifestyle practices, and psychological factors [2]. Male infertility contributes to the global decline in fertility rates, resulting in slower population growth, population decline, and significant global aging trends. Recent research highlights the prevalence of microorganisms that primarily reside in the human intestine, forming a crucial intestinal microbial system that profoundly impacts human health. These microorganisms outnumber human cells by about tenfold, totaling approximately 100 trillion cells and encoding a unique genome a hundred times larger than that of humans [3,4]. This underscores the critical role of microorganisms in human health. The gut microbiota significantly influences various aspects of human health, such as digestion and absorption in the gut, predisposition to obesity and metabolic disorders, immune system modulation, mental well-being, and even male fertility issues [5]. In particular, the gut microbiota and male reproductive health are intricately interconnected, potentially influencing immune regulation, endocrine function, neuroendocrine interactions, hormone levels, inflammatory responses, and nutrient metabolism.

The human gut harbors a vital microecosystem, primarily the intestinal microbiota, with robust metabolic capabilities that support normal bodily functions and provide protective mechanisms against viral and pathogenic invasions. The stability of the intestinal flora is crucial, as disturbances can lead to a range of intestinal and extraintestinal diseases, highlighting the importance of maintaining a balanced intestinal microecological environment to enhance resistance against infectious diseases caused by intestinal pathogens [6]. In general, the symbiotic relationship between the gut microbiota and the host organism is paramount for maintaining health in humans and other animals. The human gut microbiota comprises bacteria, archaea, eukaryotic bacteria, viruses, and parasites [7]. Bacteria dominate the intestinal microbiota, accounting for more than 99% of the total population and consisting of 500–1000 different species classified as beneficial, neutral, or harmful [8]. Beneficial bacteria contribute to vitamin synthesis, neutral bacteria support overall health, and harmful bacteria pose risks as pathogens. Research demonstrates that the intestinal microbiota exerts a significant functional role in maintaining healthy physiological activities by colonizing intestinal spaces, interacting with intestinal surfaces, and utilizing resources to hinder the growth of pathogenic microorganisms, thus safeguarding the intestines and diminishing the likelihood of infections [9]. Certain intestinal microbes synthesize essential substances such as vitamins and amino acids necessary for physiological metabolic functions [10]. Furthermore, intestinal microorganisms aid in food digestion, breaking down complex polysaccharides, generating absorbable nutrients, and improving digestion efficiency [11]. They can also facilitate chondrogenesis by participating in glycosaminoglycan biosynthesis. Gut microbes influence the immune system of the gut, regulating intestinal immunity to prevent autoimmune diseases and maintain immune balance [12]. Furthermore, the composition of intestinal microbes is correlated with metabolic disorders such as obesity and diabetes, which affects energy metabolism and fat storage [13]. These microbes also play a role in the central nervous system, influencing the mental health of the organism [14]. Various methods, such as UniFrac, assess and measure intestinal microbial diversity, offering insights into the characteristics of microbial communities. Promising advances in diagnostic and therapeutic applications emerge through innovative approaches like low-error 16S ribosomal RNA amplicon sequencing combined with whole genome sequencing, bolstering the potential of the gut microbiota as a diagnostic tool and therapeutic target (Table 1) [15].

Various studies have demonstrated the close association between the gut microbiota and male reproductive health, highlighting its multifaceted impact on male reproductive function. The intestinal microbiota significantly influences the regulation of the immune system, maintaining the equilibrium of the intestinal immune system for normal physiological functions. Disruption of this balance can cause irregularities in the immune system, which subsequently affect normal reproductive function [25,26,27]. “Balance” refers to the symbiotic relationship that exists between the host’s commensal microbiota and the host’s normal physiologic immune activity, as well as the balance between the composition and function of the microbiota. The microbiota plays a crucial role in inducing and regulating local and systemic immune responses. On the other hand, the immune system is also involved in maintaining the composition of the microbiota. Optimal microbiota-immune system cross-talk is essential for protective responses to pathogens and for immune tolerance to self and environmentally benign antigens. Any disruption of this symbiotic relationship may lead to disease susceptibility [28]. In addition, the intestinal microbiota exerts an influence on hormone synthesis, utilization, and metabolism, particularly androgens and testosterone, affecting hormone levels throughout the reproductive system and contributing to the treatment of reproductive issues [29,30]. Regarding interactions with the neuroendocrine system, the gut microbiota can modulate the gut–brain–reproductive axis through interactions with the neuroendocrine system (HPG axis), influencing reproductive function by directly impacting nerve signaling and hormone secretion [31,32]. Additional factors related to the gut microbiota and male reproductive health include malabsorption of intestinal nutrients, intestinal inflammation, and microbiota-derived metabolites that can impact healthy reproductive function. Studies have identified various bacteria within the gut microbiota related to reproductive health, capable of directly influencing reproductive hormone levels or impeding the invasion of pathogens into reproductive organs by disrupting intestinal microecosystem regulations. These bacteria can also cause aberrations in the immune, endocrine, nervous, digestive, and other interconnected systems, indirectly causing reproductive problems. Notable examples of such bacteria include *Lactobacillus* spp., *Bifidobacterium* spp., *Streptococcus* spp., *Anaplasma* spp., *Enterococcus* spp., *Clostridium* spp., and *Bacillus* spp., among others [33]. Lactobacilli, beneficial bacteria within the intestinal microbiota, regulate the balance of acid and base of the organism through lactic acid production, sustaining normal physiological functions. In the female reproductive tracts, lactobacilli aid in maintaining a vaginal acidic environment, thwarting harmful bacterial proliferation to protect reproductive health [34]. *Bifidobacterium bifidum*, an early colonizer of the human gastrointestinal tract, promotes host health and helps maintain intestinal microecological equilibrium, indirectly ensuring organismic reproductive health [35]. Bacteroidetes, beneficial intestinal microorganisms present in the intestines and various parts of the body, metabolize indigestible polysaccharides and oligosaccharides while synthesizing essential vitamins for the body and other intestinal microorganisms to sustain healthy life activities, thus indirectly promoting reproductive health [36].

## 2. Possible Mechanisms

### 2.1. Regulation of the Immune System

The immune system comprises the innate immune system and the acquired immune system, intricate defense mechanisms crucial to protecting the body against pathogens such as bacteria, viruses, and fungi, and eliminating harmful entities to prevent diseases such as cancer and autoimmune diseases [37]. The innate immune system represents the body’s intrinsic defense mechanism spread across various tissues and cells, such as skin tissues, mucous membrane tissues, natural killer cells, and macrophages. Although this system responds rapidly, its specific recognition efficiency remains relatively low, primarily serving to isolate foreign pathogens from internal body contact. On the other hand, the acquired immune system learns to recognize and defend against pathogens through exposure, classified into the T-cell immune system and the B-cell immune system. T cells identify infected cells and pathogens, whereas B cells generate antibodies to bind to recognized pathogens, expelling them from bodily fluids [38]. The immune system is vital to maintaining biological health of organisms, acting as a primary defense mechanism against pathogenic attacks and the development of diseases, including cancer. Moreover, interrelated with various body systems, any irregularities of the immune system can disrupt the equilibrium of the body, leading to the onset of diseases, such as autoimmune diseases, when the immune response becomes excessively active [39].

**Figure 1:**
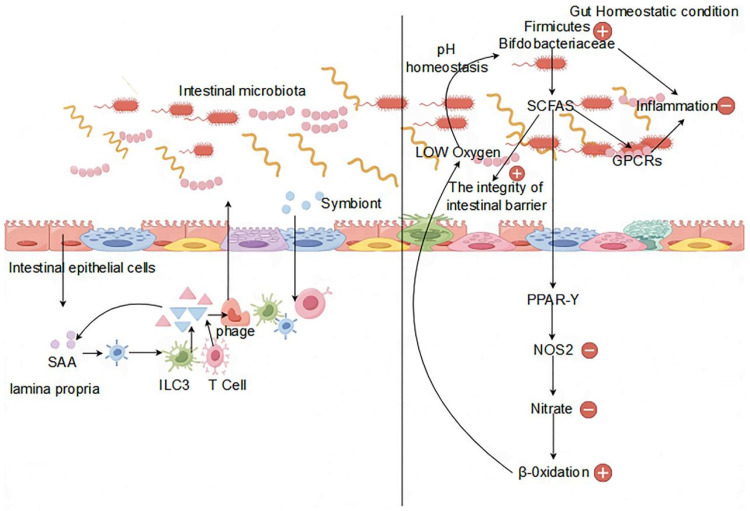
Impact of the gut microbiota on the immune system.

An imbalance in the gut microbiota disrupts the microecological environment of the gut, impacting the immune system and consequently resulting in immune dysfunction. This imbalance adversely affects multiple aspects of the immune system. Regarding immune regulation, an imbalanced intestinal microbiota hinders the activity, growth, and differentiation of T cells, B cells, and regulatory T cells, thus compromising the immune capacity and tolerance of the organism, especially affecting the mucosal immune system [40]. Regarding regulation of the inflammatory response, the imbalance of the gut microbiota inhibits the signaling pathways of immune transduction between intestinal mucosal epithelial cells and immune cells, increasing the levels of overall organismic inflammatory responses due to regulatory limitations. Furthermore, certain gut microorganisms produce anti-inflammatory agents such as short-chain fatty acids; an imbalance reduces their production, intensifying inflammation levels [41]. It has now been shown that the gut microorganisms that produce SCFA have been largely identified. For example, *Akkermansia muciniphila* is recognized as the essential bacterium for the production of propionate, which contributes to the degradation of mucins. Similarly, in the colon, *Ruminococcus bromosus* makes a significant contribution to butyrate production by fermenting resistant starch. In addition, *Faecalibacterium prausnitzii*, *Eubacterium hallii*, and *Eubacterium rectale* are also recognized as major butyrate producers. *Prevotella* spp., *Streptococcus* spp., *Lactobacillus* spp., *Bifidobacterium* spp., *Clostridium* spp., *Clostridium mucilaginousum* spp., *Clostridium hydrogenatum* spp., and *Ruminococcus* spp. produce acetate as a SCFA metabolite via the Wood–Jungdahl pathway and the pyruvate-decarboxylation to acetyl-CoA pathway [42]. *E. hallii*, *Roseburia* spp., *Anaerostipes* spp., *Coprococcus comes*, *F. prausnitzii*, *E. rectale*, *C. catus*, and *Coprococcus eutactus* produce butyric acid via exogenous acetic acid and butyric acid kinase pathways. *Zoogloea ramigera*, *Megasphaera* and *Escherichia coli* utilize thiolases to process butyryl-CoA, propionyl-CoA, and acetyl-CoA to produce valeric acid and hexanoic acid [43]. In terms of immune response, specific strains of the gut microbiota synthesize immunosuppressive agents, such as polysaccharides, lipopolysaccharides, and peptides, influencing organismic immune responses [44]. Disruption in the balance of the intestinal microbiota can alter the levels of these immunosuppressive substances, affecting the efficiency of immune function or leading to immune hyperactivity, thus resulting in immune malfunction (Figure 1).

The testicular mesenchyme exhibits potent immunomodulatory activity, influencing various immune cells such as macrophages, T cells, dendritic cells, mast cells, and others to produce cytokines, androgens, and related immunomodulatory molecules. For example, immune signaling pathways can be regulated by anti-inflammatory factors such as TGF-β and IL-10, reducing immune responses and maintaining immune balance in the testes [45,46]. These immune cells exhibit a high tolerance to germ cell autoantigens. Ongoing research has identified androgens, prostaglandins, and microenvironmental cues such as corticosterone as immunomodulatory molecules believed to shape the function and phenotype of interstitial testicular immune cells [47]. Within the organism, macrophages in the interstitium of human testes are classified into interstitial and peritubular macrophages, comprising 62% of testicular myeloid cells, with interstitial macrophages of the rat testicular interstitial macrophages constituting 80% of testicular leukocytes [48,49]. Therefore, macrophages are the predominant immune cells in the testicular interstitium [50]. Studies have shown a significant increase in testosterone production when interstitial macrophages interact with conditioned medium for testicular macrophages, highlighting the crucial role of close interactions between these cells in promoting testosterone synthesis [51]. Testicular macrophages can produce the 25-hydroxycholesterol cytokine as a substrate for testosterone synthesis, positively impacting testosterone levels [52]. In CSF1 mutant mice lacking most macrophages, testosterone levels in the testes decrease. Testosterone, which acts via the androgen receptor in Sertoli cells, regulates spermatogenesis by modulating downstream gene expression such as Rhox5, thus overseeing spermatogenesis processes, including germ cell maintenance, integrity of the blood–testis barrier, meiotic completion, adhesion of sperm to Sertoli, and sperm release [53,54]. Macrophages contribute to sperm proliferation and differentiation through the expression of the CSF1 and retinoic acid biosynthesis enzymes ALDH1A2 and RDH10, and reduced macrophages in the testicular mesenchyme alter spermatogonial differentiation [55].

In a healthy organism, lymphoid T cells constitute 10–20% of total leukocytes, spread throughout the interstitium. Unlike lymphoid B cells, lymphoid B cells are absent from the interstitial space in the testicular interstitium. Interstitial lymphocytes in the testes encompass effector T helper cell 1 (Th1), effector T cell 7 (Th17), and Tregs. In autoimmune diseases, the number of T cells in the testes increases significantly. Foxp3+ Tregs act as potent immunosuppressive cells found in rat, mouse, and human testes under physiological conditions, contributing to the immunosuppressive properties of the testes. In particular, testosterone supplementation increases the population of CD4+ CD25+ Foxp3+ Tregs, mediated by androgen-induced binding of AR to the Foxp3 locus [56]. Dendritic cells, although a minor group among testicular immune cells, play a crucial role in deactivating effector T cells, fostering Treg growth, and influencing the normal operations of the adaptive immune system in the testes. Immature dendritic cells under physiological conditions protect spermatocytes by binding antigens to normal spermatocytes, inducing tolerance. However, mature dendritic cells, when stimulated, upregulate co-stimulatory proteins and inflammatory cytokines, leading to autoimmune T-cell activation, reducing immune tolerance, and potentially causing male reproductive sterility [57].

In the testes, a variety of immune cells have been identified, each with unique metabolic processes and mechanisms that play a critical role in their phenotype and regulation of plasticity [58]. Inflammatory stimuli activate and polarize macrophages, leading to metabolic reprogramming that shifts normal mitochondrial metabolism towards ROS production, favoring glycolytic pathways. This change positively affects the transcription of the pro-inflammatory cytokine IL-1β, known to reduce testicular steroid production, and IL-1β itself, which positively influences autocrine cell regulation [59]. The relationship to the autocrine regulatory function of cells is noteworthy. Different immune cells undergo different metabolic processes and produce various metabolites in different external and internal immune stimulation states. Metabolic processes not only enable macrophages to produce immune cytokines but also allow other immune cells to generate different immune factors with diverse functions. Immune cells can impact cytokine levels through metabolic processes, whereas cytokines can reciprocally influence immune cell function and metabolism. Activation of an immune cell by a relevant stimulus triggers changes in cell metabolism and immune cytokine production, ultimately disrupting the normal immune system function of the testes [60].

### 2.2. Effects of the Endocrine System

In the human body, the endocrine system serves as a vital regulatory mechanism, responsible for secreting various hormones into the bloodstream to influence physiological functions and cellular activities throughout the body [61]. This intricate system comprises several essential endocrine glands, including the hypothalamus, thyroid, pancreas, pituitary glands, adrenal glands, and gonads. The hypothalamus, located at the base of the brain, regulates hormone release and influences the secretory activities of the pituitary gland [62]. The thyroid, located in the front of the neck, produces thyroxine and triiodothyronine, which are crucial for basal metabolism and energy balance. Likewise, the pancreas, located in the abdominal cavity, releases insulin and glucagon, which are essential for maintaining blood glucose homeostasis. The pituitary glands, located at the base of the brain, secrete a variety of hormones, such as growth hormone, thyroid hormone, and adrenocorticotropic hormone, essential for normal physiological functions [63]. Positioned above the kidneys, the adrenal glands secrete adrenocorticotropic and adrenomedullary hormones, which affect metabolic activities and immune functions. The gonads, which include the testes and ovaries, produce androgens and estrogens, such as testosterone and progesterone, crucial for the regulation of reproductive physiology [64]. In general, the hormones produced by the endocrine system are essential to regulate metabolic activities, growth, development, reproductive functions, and internal environment homeostasis. Interacting with the nervous system, the hypothalamus acts as a key mediator within the endocrine system, which maintains hormone levels through negative feedback mechanisms. Any dysregulation within the endocrine system can alter the internal homeostasis of the organism, leading to various physiological abnormalities and diseases, such as diabetes mellitus, hyperthyroidism, abnormal adrenal function, and sexual dysfunction [65].

The imbalance of the intestinal microbiota disrupts the homeostasis of the internal environment of the body, affecting various physiological processes, especially the endocrine system. This imbalance leads to abnormalities in the immune system, which affects the immune function of the body, which is closely interlinked with the endocrine system. Dysregulation of the intestinal microbiota initiates immune dysfunction, subsequently influencing the stability of the endocrine system. Under normal conditions, the intestinal barrier protects the mucosa of the microbiota and their metabolites. However, immune dysfunction caused by the microbiota increases the inflammatory response in the intestines, increasing mucosal permeability. This increased permeability allows the easier entry of harmful substances into the internal environment, ultimately affecting the endocrine organs through the bloodstream, thus compromising endocrine function [66,67]. The endocrine system relies on hormones secreted by endocrine glands to maintain normal physiological functions of the body. Some intestinal microbiota are intricately connected to the metabolism and physiological effects of relevant hormones in the body. For example, the microbiota influence sex hormone levels such as testosterone and progesterone, disrupting the negative feedback regulation loop of the endocrine system and leading to abnormal secretion of sex hormones, consequently affecting reproductive function [68]. Gut microbes produce anti-inflammatory substances such as short-chain fatty acids, which modulate hormone sensitivity, such as insulin, in the digestive process, affecting the immune and endocrine systems. In summary, when the balance of the gut microbiota is compromised, the endocrine system is directly or indirectly affected, resulting in endocrine disorders.

**Figure 2:**
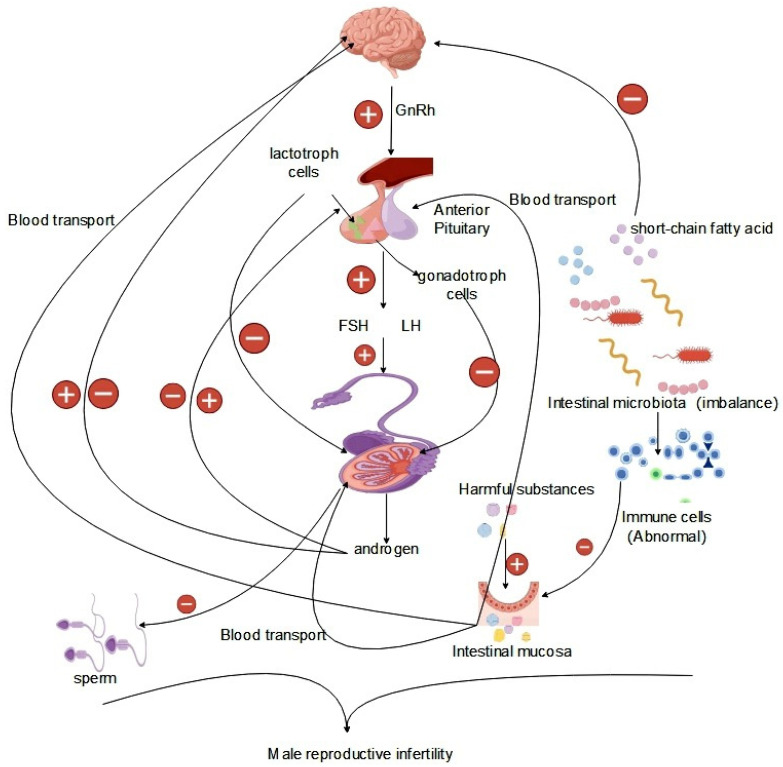
Effect of the gut microbiota on the HPG axis.

The hypothalamic–pituitary–gonadal (HPG) axis, vital for the regulation of body growth, development, and the reproductive system, plays a critical role [69]. Within the HPG axis, the hypothalamus orchestrates the pulsatile release of gonadotropin-releasing hormone (GnRH), which activates the pituitary–gonadal axis. GnRH stimulates the pituitary gland to produce luteinizing hormone (LH) and follicle-stimulating hormone (FSH), which are crucial for male reproductive processes. LH regulates intertesticular cell function and testosterone secretion, while FSH promotes germ cell division and sperm production, as well as supporting energy metabolism in testicular germ cells. Testosterone, through negative feedback, inhibits gonadotropin secretion, ensuring hormonal balance in the reproductive system [70]. These hormones maintain internal environmental homeostasis in the reproductive system, which is crucial for healthy physiological activities. On the HPG axis, hormones facilitate spermatogenesis and regulate the quantity and quality of sperm [71,72]. During testicular metabolism, the energy requirements of germ cells within the testis are addressed by support cells, which preferentially utilize lactic acid secreted by cells as the raw material for the generation of ATP through the glycolytic pathway and the use of the mitochondrial fatty acid oxidation pathway to satisfy the energy requirements of germ cells for normal physiological processes [73]. Germ cell energy needs are met by support cells using lactic acid, glucose, and lipids. FSH regulates energy substance uptake, glycolytic metabolism, and lipids, which affects support cell metabolism [74,75,76]. Androgens and estrogens regulate testicular support cell metabolism, influencing glycemic absorption and lactic acid production [77]. The metabolic processes involved in the development and functioning of reproductive cells require energy metabolism. For example, LH regulates cholesterol metabolism to produce testosterone and other steroid hormones in testicular cells, involving energy expenditure [78]. Hormone synthesis on the HPG axis is dependent on endocrine system activity, particularly testosterone and steroid hormones, vital for sperm production and normal male reproductive function (Figure 2).

Studies have shown that metabolic disorders of the endocrine system can induce a variety of abnormalities in male reproductive function. The endocrine system of the human body comprises various endocrine glands, such as the gonads (testes, ovaries), adrenal glands, thyroid gland, hypothalamus, pituitary gland, and pancreas [79]. These endocrine glands are interconnected and the hormones they secrete interact with each other. Dysfunction in the hypothalamus, pituitary gland, and HPG axis gonads, as well as disorders of the adrenal glands, thyroid gland, and pancreatic endocrine glands, can lead to reproductive abnormalities in men. Specifically, disturbances in gonadal function can affect the secretion of testosterone hormone by testicular interstitial cells, affecting spermatogenesis and the overall growth and development of the organism [80]. Dysfunction in the hypothalamus can alter the levels of prohormone-releasing hormone and regulatory hormones, causing an imbalance in the levels of hormones of the body, which in turn impairs normal reproductive function. In cases of pituitary gland dysfunction, changes in prohormone levels, particularly luteinizing hormone (LH) and follicle-stimulating hormone (FSH), can disrupt sex hormone secretion, affecting sperm production and quality through the negative feedback mechanism of the endocrine system. Adrenal dysfunction can result in chronic overstimulation, leading to excessive cortisol secretion, which in turn interferes with hormone production by the testes and affects male reproductive function [81]. Thyroid gland dysfunction can cause abnormalities in thyroid hormone secretion, leading to hyperthyroidism or hypothyroidism, both of which can alter normal hormone production by the testes, affecting sex hormone secretion and sperm quality [82]. Pancreatic dysfunction can lead to uncontrolled levels of insulin and glucagon, which can cause diabetes and obesity. In cases of diabetes, glucose uptake and utilization in testicular cells, as well as energy metabolism processes, are affected, ultimately affecting spermatogenesis [83]. Obesity can alter lipid metabolism, increase estrogen production by adipocytes, reduce testosterone and LH levels, and consequently decrease sperm production and quality, affecting male reproductive health [84].

### 2.3. Interaction of the Gut–Brain–Reproductive Axis

The concept of the brain–gut microbiome axis, which facilitates bidirectional communication between the gut, the gut microbiota, and the nervous system, has been elucidated in relevant studies [85,86]. Experimental animal studies have confirmed the regulatory role of the gut microbiota in the organism, and below, we elaborate on the mechanisms of the gut–brain axis using various research examples. The key to the association of the gut microbiota with the central nervous system (CNS) are intermediates generated by the gut microbiota, including short-chain fatty acids, secondary bile acids, and tryptophan metabolites [87,88]. Tryptophan metabolites produced by the gut microbiota signal to the CNS through multiple pathways, enhancing glucose metabolism by generating FGF19, suppressing the HPA axis by producing FGF19, and promoting the release of GLP-1 and PYY from L cells through the TGR5 receptor [89,90]. The production of short-chain fatty acids leads to the production of leptin by adipocytes via GPR41, after transversely crossing the CNS [91]. Similarly, the synthesis of secondary bile acids triggers the production of CCK from EECs through tlr signaling through the CNS [92]. Intermediates can directly convey signals to high levels through interactions with the intestinal mucosal system, enteroendocrine cells, and enterochromaffin cells, or they can traverse the intestinal mucosal barrier into the bloodstream, subsequently acting at specific sites to transmit signals to higher levels. Gut microbes release microbial signals that communicate directly via vagal pathways and transmit signals from the spinal cord to nerves [93,94].

Numerous studies indicate a close relationship between the gut microbiota and immune signaling in the nervous system. Studies in germ-free mice and mouse models exposed to broad-spectrum antibiotics have revealed that disruptions in gut microbiota neuromodulatory signaling lead to abnormalities in neurodevelopment and neurological disorders in mice, underlining the intimate connection between the gut microbiota and the mouse nervous system [95,96]. The development of microglia is regulated by both the gut microbiota and the CNS. Microglia are brain tissue macrophages that comprise 10–15% of brain macrophages, playing crucial roles in CNS development; early stage antigen presentation; direct communication with neurons, astrocytes, and blood vessels through cell body extensions; and regulation of inflammation. Dysregulation of the microbiota results in altered microglial levels, inflammatory diseases, altered organism homeostasis, and neurological abnormalities [97,98]. Short-chain fatty acids synthesized by the gut microbiota contribute to the growth, maturation, and maintenance of normal physiological functions of microglia, highlighting the significance of the gut microbiome and the gut–brain axis in microglia development studies. In essence, alterations in the gut microbiota affect the functional activities of the CNS, while reciprocal changes in the CNS affect the balance of the gut microbiota.

**Figure 3:**
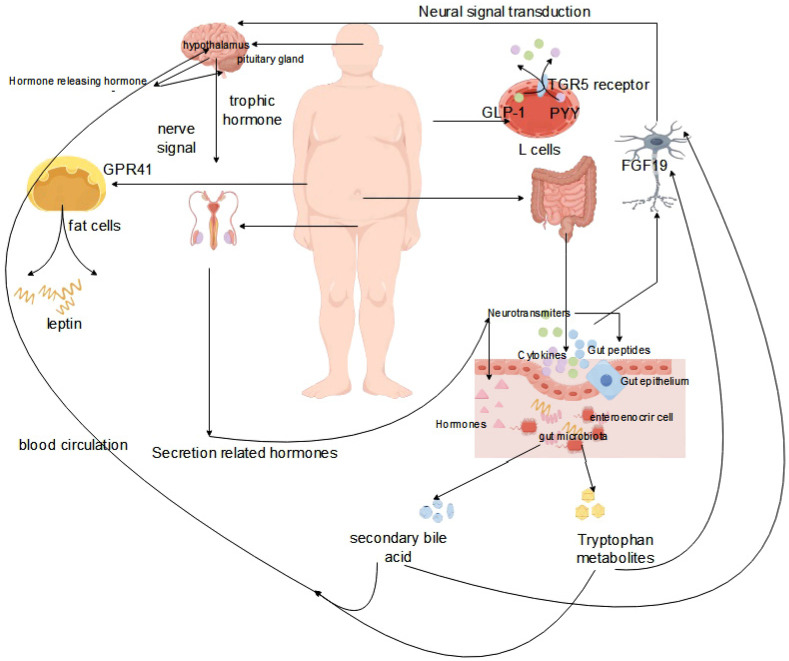
Effect of the gut microbiota on the interactions of the gut–brain–reproductive axis.

In the study of the HPG axis, it is important to understand that this axis comprises the hypothalamus, pituitary gland, and gonads organized sequentially, thus forming the hypothalamic–pituitary–gonadal axis (HPG) [99,100]. The HPG axis functions by releasing hormones in response to signals from the nervous system, making it a crucial neuroendocrine system that primarily regulates various physiological activities, particularly those related to growth and reproduction. Situated deep within the base of the brain, the hypothalamus not only ensures bodily stability, but also serves as a vital link between the nervous and endocrine systems [101]. Through input from the neural and peripheral nervous system, the hypothalamus acts as the central control system, adjusting hormone levels to maintain normal physiological functions even in the face of failure. Hormones within the HPG axis act as chemical messengers that fine-tune the functioning of different body parts upon release into the bloodstream, facilitating optimal organism performance. Neurons stimulate the hypothalamus to secrete gonadotropin-releasing hormone (GnRH), which triggers the pituitary gland to release follicle-stimulating hormone (FSH) and luteinizing hormone (LH), crucial for testosterone regulation in men and sperm production. The pituitary gland, located below the hypothalamus, plays a pivotal role in hormone secretion, including adrenocorticotropic hormone (ACTH), growth hormone (GH), and thyroid stimulating hormone (TSH), all controlled by hypothalamic signals. In particular, the secretion of LH and FSH is essential for maintaining male reproductive functions. The GnRH released by the hypothalamus is vital for the final release of sex hormones through multiple neuronal and epigenetic regulations. Recent research has highlighted the strong connection between the hypothalamic–pituitary axis and the central nervous system [102,103], highlighting the intricate relationship and the potential wider implications of the neuroendocrine–HPG axis. Kisspeptin neurons in the hypothalamus, crucial for the regulation of reproductive function, are located centrally in both the anterior and posterior regions and play a key role in the secretion of pulsatile gonadotropin-releasing hormones. Phoenixin (PNX) has emerged as a significant neuropeptide that affects gonadotropin-releasing hormone secretion on the HPG axis, particularly by regulating GnRH receptor expression in the pituitary gland. Excess PNX secretion leads to decreased levels of gonadotropin, negatively affecting sperm production and related reproductive functions [104] (Figure 3).

## 3. Intervention and Treatment Strategies

Male infertility is usually associated with microbiota dysbiosis, an imbalance in the composition and function of the microbiota. This imbalance is thought to affect infertility through various mechanisms. Inflammation and immune response: One important mechanism linking microbiota dysbiosis to male infertility is the activation of inflammation and immune response. In the case of male infertility, inflammation and immune response can negatively affect sperm function and overall fertility, and ROS produced by the body during inflammation can induce OS. It has now been shown that OS impairs the fluidity of the sperm plasma membrane and the integrity of the DNA, leading to reduced sperm counts and impaired sperm function, which can negatively affect fertility. OS and its effect on sperm quality: OS is an imbalance between ROS production and the body’s antioxidant defenses, and it has been linked to male infertility [105]. Although ROS are essential for reproduction, their overproduction can damage sperm DNA, impair sperm viability, and increase susceptibility to genetic abnormalities. ROS alter sperm morphology, decrease sperm concentration, and affect overall semen parameters. Mechanisms by which OS affects sperm quality include lipid peroxidation, DNA damage, and impaired mitochondrial function. Mitochondrial function is critical for sperm motility because mitochondria provide the energy required for sperm movement. Sperm motility is heavily dependent on mitochondrial function, as these organelles play a crucial role in providing the necessary energy for sperm motility. Impaired mitochondrial function reduces the production of adenosine triphosphate (ATP), resulting in decreased sperm motility and fertility [106]. Impaired sperm function and viability: Sperm motility and dysfunction are common in male infertility. The composition of the gut microbiota is influenced by factors such as diet and the immune system, which can affect sperm function and viability. Dysbiosis of the gut microbiota, characterized by reduced microbial diversity and growth of specific bacterial taxa, has been associated with impaired sperm function and motility. Factors such as oxidative stress, phage induction, and bacterial toxin release can trigger this dysbiosis [107]. Notably, pathogenic bacteria can also negatively affect sperm function and viability. Bacterial infections in the male reproductive tract can induce inflammation and oxidative stress, affecting sperm quality and overall fertility. The presence of intracellular bacteria in the male reproductive tract can trigger an immune response that interferes with sperm function, leading to fertility problems [108].

Dysbiosis of the intestinal microbiota can cause a variety of disruptions in bodily functions and metabolic processes, as well as abnormal system activities, increasing the risk of disease. Thus, preventing intestinal microorganism dysbiosis is crucial to maintaining normal physiological functions. As a general guideline, a balanced diet is recommended that includes an abundance of vegetables, fruits, grains, and cereals rich in dietary fiber. These foods can encourage the growth and proliferation of beneficial bacteria in the intestinal microbiota. Careful cleaning of food surfaces is essential to prevent pesticide residues from entering the digestive tract, potentially causing the demise of intestinal flora and triggering dysbiosis of intestinal microflora. It is recommended to avoid pesticides such as thiamethoxam and butachlor [109,110,111,112,113,114]. When using medications for common diseases, it is important to limit the excessive use of disinfectants and bactericides to avoid disrupting the intestinal microbiota. Similarly, overuse of antibiotics should be avoided as they can destroy beneficial intestinal microorganisms, compromising the stability of the microbiota. It is recommended to practice good personal hygiene using mild detergents and avoiding strong antibacterial products to maintain intestinal microbiota balance. Consumption of probiotic- and prebiotic-rich beverages, containing live beneficial bacteria and food ingredients that support probiotic growth, respectively, is encouraged [115,116,117]. Furthermore, moderating smoking and alcohol consumption, ensuring adequate sleep, regular exercise, and stress management all contribute positively to preventing intestinal microbiota dysbiosis.

**Figure 4:**
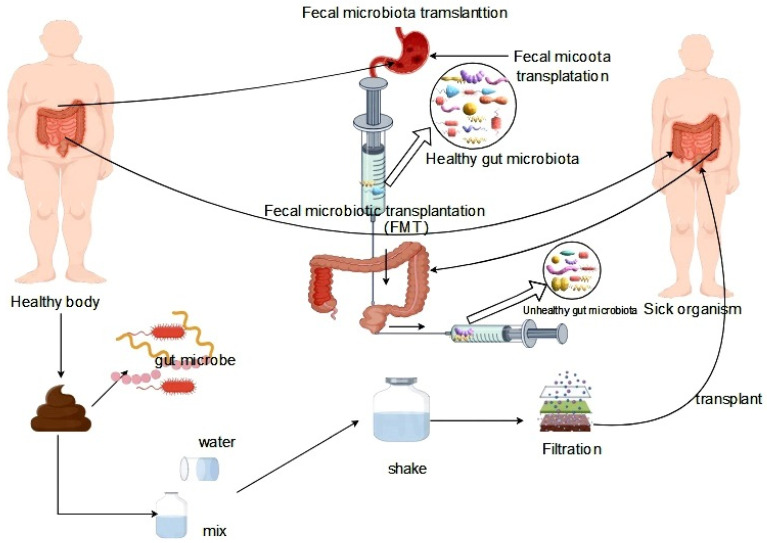
Fecal transplantation for the treatment of gut microbiota dysbiosis.

The principles of treating intestinal microbiota dysbiosis can be classified into microbial therapy and pharmacotherapy. Microbial therapy comprises probiotic therapy and fecal transplantation therapy. Probiotics are commonly included in daily diets and offer numerous benefits in maintaining healthy metabolic functions, such as promoting intestinal health and regulating intestinal microbiota homeostasis. Probiotics can inhibit the growth and reproduction of pathogenic microorganisms in the intestinal tract and ferment into various intestinal metabolites, such as short-chain fatty acids (SCFA) such as acetate, butyrate, and propionate. These metabolites have favorable effects on the balance of *Lactobacillus*, *Bifidobacterium*, and other bacteria in intestinal microbiota homeostasis, promoting the growth and reproduction of beneficial bacteria [118,119]. Fecal transplantation therapy (FMT) is a therapeutic strategy that involves transferring fecal material from a healthy donor to a recipient in order to reprogram the regulation of the gut microbiota, with the objective of stabilizing the structural composition of the gut microbiota and normalizing its function. The specific process of FMT includes the transfer of the intestinal microbiota from a healthy organism to the intestinal tract of a diseased individual, which corrects intestinal microbiota disorders within the recipient and treats the disease [120] (Figure 4).

Drug therapy aimed at regulating intestinal microbial balance is a prominent focus in current medical research. Among the limited drugs used to preserve the intestinal microbiota, the Gannetna capsule (code name: GV-971) stands out. This capsule targets the brain–gut axis, effectively restoring the intestinal microbiota; reducing abnormal concentrations of amino acids in both blood and feces; and mitigating neuroinflammation associated with peripheral helper T cells, particularly Th1 cells in the brain. Furthermore, compound herbs have shown the potential to maintain intestinal microbiota homeostasis by influencing the structure, composition, and function of the intestinal flora [121]. Both microbial and pharmacological treatments play a pivotal role in the balance of intestinal microorganisms, leading to a lower prevalence of the disease and ensuring optimal metabolic activity in the body.

## 4. Challenges and Future Research Directions

The study of male reproductive infertility mechanisms triggered by gut microbiota dysbiosis has limitations, predominantly due to the use of animal models in research with limited human evidence. The divergence between the gut microbiota in animals and humans restricts the broad applicability of the study results. Although some research has suggested a link between gut microbiota dysbiosis and male infertility, the evidence does not establish a conclusive causal relationship, given the influence of various physiological and external factors on male infertility. Insufficient research has been conducted on drug treatments for GI dysbiosis, specifically in regard to male infertility caused by GI dysbiosis. This inadequacy underscores the limitations of drug use for this purpose.

The current focus of biomedical research is the link between the gut microbiota and male reproductive infertility. To improve our understanding of how gut microbiota dysbiosis impacts male reproductive infertility mechanisms, in-depth analyses of gut microbiota composition and diversity are imperative. Variations in the gut microbiota between individuals and specific microorganism content may significantly impact male reproductive sterility. More research is essential to investigate how the gut microbiota influences the activities of the immune and reproductive systems, contributing to male reproductive abnormalities. Exploring these interactions is crucial for studying drugs used to treat men’s reproductive health issues.

For future studies on the impact of the gut microbiota on male reproductive infertility, several key areas should be emphasized. First, the establishment of systematic animal models that closely mirror the mechanisms of the human gut and the composition of the microbiota will elucidate how changes in the gut microbiota lead to male reproductive sterility. Second, the focus should shift to understanding the regulatory mechanisms of the body’s immune system. By exploring the intricate interplay between the gut microbiota and the immune system, a deeper understanding of how gut microbiota imbalances disrupt immune system homeostasis and impact male reproductive function can be achieved. Lastly, integrating various scientific research methods such as molecular biology, molecular biochemistry, immunology, and reproductive studies to investigate the relationship of the gut microbiota with male reproductive infertility will provide a comprehensive understanding of the mechanisms, thereby laying a theoretical foundation for the treatment and prevention of male reproductive issues.

## 5. Conclusions

In conclusion, the gut microbiota plays a vital role in maintaining normal reproductive function in organisms by closely interacting with the body’s immune system. This symbiotic relationship ensures that the immune system’s metabolic activity functions effectively to ward off pathogens, safeguarding the reproductive system, and upholding overall reproductive health. A thriving gut microbiota also supports male sperm production and quality, positively impacting fertility. Certain metabolites produced by intestinal microorganisms can trigger inflammation, such as orchitis, which can affect fertility. Therefore, preserving a healthy intestinal microbiota is crucial for the health of male reproductive organs. Promoting intestinal health through a balanced diet, healthy lifestyle choices, and judicious medication use is essential to maintain the equilibrium of the intestinal microbiota, protect the male reproductive system, and promote its growth and development.

The significance of investigating the relationship between the gut microbiota and male reproductive infertility lies in elucidating how a healthy gut microbiota enhances male fertility, particularly in spermatogenesis and sperm quality. On the contrary, imbalances in the gut microbiota can negatively impact reproductive health. This underscores the crucial role of the gut microbiota in male reproductive processes. Research on how the gut microbiota influences male reproductive health aims to explicate the entire chain of influence and establish a theoretical foundation for future studies on drugs targeting gut microbiota imbalance and male reproductive disorders. This research is of significant importance for clinical applications.

## Figures and Tables

**Table 1:** Classification, composition, and function of the major gut microbiota.

Classification	Composition	Function
Bacteria	Probiotics	Maintain intestinal pH balance, inhibit pathogen growth and reproduction, and promote digestion and absorption [16].
	Lactobacilli	Reduce intestinal pH value, inhibit the growth of harmful bacteria [17].
	Thick-walled bacteria	Influence the degradation and absorption of intestinal food [18].
	Morphogenic bacteria	Plays an important role in the carbohydrate metabolism process in the intestinal tract [19].
	Enterococci	Regulate intestinal health and the immune system [20].
	Actinomycetes	Synthesize antibiotics [21].
	*Streptococcus* faecalis	Short-chain fatty acids and other metabolites that contribute to maintaining homeostasis in the intestinal environment [22].
Fungi	Yeasts	Assist in the intestinal digestive process [23].
Viruses	Poliovirus	Causes intestinal infections, leading to an immune response [24].

## References

[1] Barbu M.G., Thompson D.C., Suciu N., Voinea S.C., Cretoiu D., Predescu D.V. (2021). The Roles of MicroRNAs in Male Infertility. Int. J. Mol. Sci..

[2] Esteves S.C., Humaidan P. (2023). Towards infertility care on equal terms: A prime time for male infertility. Reprod. Biomed. Online.

[3] Backhed F., Ley R.E., Sonnenburg J.L., Peterson D.A., Gordon J.I. (2005). Host-bacterial mutualism in the human intestine. Science.

[4] Hooper L.V., Midtvedt T., Gordon J.I. (2002). How host-microbial interactions shape the nutrient environment of the mammalian intestine. Annu. Rev. Nutr..

[5] Sheykhsaran E., Abbasi A., Ebrahimzadeh L.H., Sadeghi J., Mehri S., Naeimi M.F., Feizi H., Bannazadeh B.H. (2023). Gut microbiota and obesity: An overview of microbiota to microbial-based therapies. Postgrad. Med. J..

[6] Xu J., Chen H.B., Li S.L. (2017). Understanding the Molecular Mechanisms of the Interplay Between Herbal Medicines and Gut Microbiota. Med. Res. Rev..

[7] Al B.Z., Nitert M.D., Mousa A., Naderpoor N. (2020). The Gut Microbiota and Inflammation: An Overview. Int. J. Environ. Res. Public Health.

[8] Jin M., Qian Z., Yin J., Xu W., Zhou X. (2019). The role of intestinal microbiota in cardiovascular disease. J. Cell Mol. Med..

[9] Jabeen Z., Bukhari S.A., Malik S.A., Hussain G., Kamal S. (2023). Improved Gut Microbiota Escalates Muscle Function Rehabilitation and Ameliorates Oxidative Stress Following Mechanically Induced Peripheral Nerve Injury in Mice. Pak. Vet. J..

[10] Maynard C.L., Elson C.O., Hatton R.D., Weaver C.T. (2012). Reciprocal interactions of the intestinal microbiota and immune system. Nature.

[11] Zhang D., Liu J., Cheng H., Wang H., Tan Y., Feng W., Peng C. (2022). Interactions between polysaccharides and gut microbiota: A metabolomic and microbial review. Food Res. Int..

[12] Day A.S., Keenan J.I., Tannock G.W. (2020). The intestinal microbiota in health and disease. J. R. Soc. N. Z..

[13] Liu B.N., Liu X.T., Liang Z.H., Wang J.H. (2021). Gut microbiota in obesity. World J. Gastroenterol..

[14] Verma H., Phian S., Lakra P., Kaur J., Subudhi S., Lal R., Rawat C.D. (2020). Human Gut Microbiota and Mental Health: Advancements and Challenges in Microbe-Based Therapeutic Interventions. Indian J. Microbiol..

[15] Faith J.J., Guruge J.L., Charbonneau M., Subramanian S., Seedorf H., Goodman A.L., Clemente J.C., Knight R., Heath A.C., Leibel R.L. (2013). The long-term stability of the human gut microbiota. Science.

[16] Duan H., Yu L., Tian F., Zhai Q., Fan L., Chen W. (2020). Gut microbiota: A target for heavy metal toxicity and a probiotic protective strategy. Sci. Total Environ..

[17] Shen X., Xie S., Zhang H., Wang T., Zhang B., Zhao H. (2023). Effects of Persimmon (*Diospyros kaki* L. cv. Mopan) Polysaccharide and Their Carboxymethylated Derivatives on *Lactobacillus* Strains Proliferation and Gut Microbiota: A Comparative Study. Int. J. Mol. Sci..

[18] Soheilian-Khorzoghi M., Rezasoltani S., Moheb-Alian A., Yadegar A., Rostami-Nejad M., Azizmohammad-Looha M., Verma A.K., Haddadi A., Dabiri H. (2022). Impact of Nutritional Profile on Gut Microbiota Diversity in Patients with Celiac Disease. Curr. Microbiol..

[19] Fehily S.R., Basnayake C., Wright E.K., Kamm M.A. (2021). The gut microbiota and gut disease. Intern. Med. J..

[20] Xu W., Fang Y., Zhu K. (2024). Enterococci facilitate polymicrobial infections. Trends Microbiol..

[21] Binda C., Lopetuso L.R., Rizzatti G., Gibiino G., Cennamo V., Gasbarrini A. (2018). Actinobacteria: A relevant minority for the maintenance of gut homeostasis. Dig. Liver Dis..

[22] Xu W., Zou K., Zhan Y., Cai Y., Zhang Z., Tao X., Qiu L., Wei H. (2022). *Enterococcus* faecium GEFA01 alleviates hypercholesterolemia by promoting reverse cholesterol transportation via modulating the gut microbiota-SCFA axis. Front. Nutr..

[23] Ianiro G., Bruno G., Lopetuso L., Beghella F.B., Laterza L., D’Aversa F., Gigante G., Cammarota G., Gasbarrini A. (2014). Role of yeasts in healthy and impaired gut microbiota: The gut mycome. Curr. Pharm. Des..

[24] Cabrerizo M., Grupo Para El Estudio de Las Infecciones Por Enterovirus Y Parechovirus (2017). Importance of enteroviruses in neuropaediatrics: From polioviruses to other enteroviruses. Rev. Neurol..

[25] de Theije C.G., Wopereis H., Ramadan M., van Eijndthoven T., Lambert J., Knol J., Garssen J., Kraneveld A.D., Oozeer R. (2014). Altered gut microbiota and activity in a murine model of autism spectrum disorders. Brain Behav. Immun..

[26] Yan J., Herzog J.W., Tsang K., Brennan C.A., Bower M.A., Garrett W.S., Sartor B.R., Aliprantis A.O., Charles J.F. (2016). Gut microbiota induce IGF-1 and promote bone formation and growth. Proc. Natl. Acad. Sci. USA.

[27] Hotamisligil G.S. (2017). Foundations of Immunometabolism and Implications for Metabolic Health and Disease. Immunity.

[28] Riazi-Rad F., Behrouzi A., Mazaheri H., Katebi A., Ajdary S. (2021). Impact of gut microbiota on immune system. Acta Microbiol. Immunol. Hung..

[29] Calcaterra V., Rossi V., Massini G., Regalbuto C., Hruby C., Panelli S., Bandi C., Zuccotti G. (2022). Precocious puberty and microbiota: The role of the sex hormone-gut microbiome axis. Front. Endocrinol..

[30] Meng J., Greenlee A.R., Taub C.J., Braun R.E. (2011). Sertoli cell-specific deletion of the androgen receptor compromises testicular immune privilege in mice. Biol. Reprod..

[31] Plant T.M. (2015). 60 Years of Neuroendocrinology: The hypothalamo-pituitary-gonadal axis. J. Endocrinol..

[32] Li X., Cheng W., Shang H., Wei H., Deng C. (2022). The Interplay between Androgen and Gut Microbiota: Is There a Microbiota-Gut-Testis Axis. Reprod. Sci..

[33] Li Y., Mehmood K., Chang Y.F., Guo R., Shang P., Zhang H. (2021). Antibiotic resistance genes in *Bacillus cereus* isolated from wild Pere David’s deer (*Elaphurus davidianus*). J. Infect..

[34] Reid G. (2008). Probiotic Lactobacilli for urogenital health in women. J. Clin. Gastroenterol..

[35] O’Callaghan A., van Sinderen D. (2016). Bifidobacteria and Their Role as Members of the Human Gut Microbiota. Front. Microbiol..

[36] Zafar H., Saier M.J. (2021). Gut Bacteroides species in health and disease. Gut Microbes.

[37] Cui B., Lin H., Yu J., Yu J., Hu Z. (2019). Autophagy and the Immune Response. Adv. Exp. Med. Biol..

[38] Eberl G., Pradeu T. (2018). Towards a General Theory of Immunity?. Trends Immunol..

[39] Place D.E., Kanneganti T.D. (2020). The innate immune system and cell death in autoinflammatory and autoimmune disease. Curr. Opin. Immunol..

[40] Gai X., Wang H., Li Y., Zhao H., He C., Wang Z., Zhao H. (2021). Fecal Microbiota Transplantation Protects the Intestinal Mucosal Barrier by Reconstructing the Gut Microbiota in a Murine Model of Sepsis. Front. Cell Infect. Microbiol..

[41] Akhtar M., Chen Y., Ma Z., Zhang X., Shi D., Khan J.A., Liu H. (2022). Gut microbiota-derived short chain fatty acids are potential mediators in gut inflammation. Anim. Nutr..

[42] Morrison D.J., Preston T. (2016). Formation of short chain fatty acids by the gut microbiota and their impact on human metabolism. Gut Microbes.

[43] Yuille S., Reichardt N., Panda S., Dunbar H., Mulder I.E. (2018). Human gut bacteria as potent class I histone deacetylase inhibitors in vitro through production of butyric acid and valeric acid. PLoS ONE.

[44] Mohr A.E., Crawford M., Jasbi P., Fessler S., Sweazea K.L. (2022). Lipopolysaccharide and the gut microbiota: Considering structural variation. Febs Lett..

[45] Fijak M., Meinhardt A. (2006). The testis in immune privilege. Immunol. Rev..

[46] Zhao S., Zhu W., Xue S., Han D. (2014). Testicular defense systems: Immune privilege and innate immunity. Cell Mol. Immunol..

[47] Wang M., Fijak M., Hossain H., Markmann M., Nusing R.M., Lochnit G., Hartmann M.F., Wudy S.A., Zhang L., Gu H. (2017). Characterization of the Micro-Environment of the Testis that Shapes the Phenotype and Function of Testicular Macrophages. J. Immunol..

[48] Ponte R., Dupuy F.P., Brimo F., Mehraj V., Brassard P., Belanger M., Yurchenko E., Jenabian M.A., Bernard N.F., Routy J.P. (2018). Characterization of myeloid cell populations in human testes collected after sex reassignment surgery. J. Reprod. Immunol..

[49] Wang J., Wreford N.G., Lan H.Y., Atkins R., Hedger M.P. (1994). Leukocyte populations of the adult rat testis following removal of the Leydig cells by treatment with ethane dimethane sulfonate and subcutaneous testosterone implants. Biol. Reprod..

[50] Winnall W.R., Hedger M.P. (2013). Phenotypic and functional heterogeneity of the testicular macrophage population: A new regulatory model. J. Reprod. Immunol..

[51] Yee J.B., Hutson J.C. (1985). Effects of testicular macrophage-conditioned medium on Leydig cells in culture. Endocrinology.

[52] Nes W.D., Lukyanenko Y.O., Jia Z.H., Quideau S., Howald W.N., Pratum T.K., West R.R., Hutson J.C. (2000). Identification of the lipophilic factor produced by macrophages that stimulates steroidogenesis. Endocrinology.

[53] O’Hara L., Smith L.B. (2015). Androgen receptor roles in spermatogenesis and infertility. Best. Pract. Res. Clin. Endocrinol. Metab..

[54] Toocheck C., Clister T., Shupe J., Crum C., Ravindranathan P., Lee T.K., Ahn J.M., Raj G.V., Sukhwani M., Orwig K.E. (2016). Mouse Spermatogenesis Requires Classical and Nonclassical Testosterone Signaling. Biol. Reprod..

[55] DeFalco T., Potter S.J., Williams A.V., Waller B., Kan M.J., Capel B. (2015). Macrophages Contribute to the Spermatogonial Niche in the Adult Testis. Cell Rep..

[56] Walecki M., Eisel F., Klug J., Baal N., Paradowska-Dogan A., Wahle E., Hackstein H., Meinhardt A., Fijak M. (2015). Androgen receptor modulates Foxp3 expression in CD4+CD25+Foxp3+ regulatory T-cells. Mol. Biol. Cell.

[57] Wang P., Duan Y.G. (2016). The role of dendritic cells in male reproductive tract. Am. J. Reprod. Immunol..

[58] Van den Bossche J., Baardman J., Otto N.A., van der Velden S., Neele A.E., van den Berg S.M., Luque-Martin R., Chen H.J., Boshuizen M.C., Ahmed M. (2016). Mitochondrial Dysfunction Prevents Repolarization of Inflammatory Macrophages. Cell Rep..

[59] Huleihel M., Lunenfeld E. (2002). Involvement of intratesticular IL-1 system in the regulation of Sertoli cell functions. Mol. Cell Endocrinol..

[60] Bhushan S., Meinhardt A. (2017). The macrophages in testis function. J. Reprod. Immunol..

[61] Murakami G., Tanabe N., Ishii H.T., Ogiue-Ikeda M., Tsurugizawa T., Mukai H., Hojo Y., Takata N., Furukawa A., Kimoto T. (2006). Role of cytochrome p450 in synaptocrinology: Endogenous estrogen synthesis in the brain hippocampus. Drug Metab. Rev..

[62] Rachdaoui N., Sarkar D.K. (2017). Pathophysiology of the Effects of Alcohol Abuse on the Endocrine System. Alcohol. Res..

[63] Ellsworth B.S., Stallings C.E. (2018). Molecular Mechanisms Governing Embryonic Differentiation of Pituitary Somatotropes. Trends Endocrinol. Metab..

[64] Estermann M.A., Major A.T., Smith C.A. (2020). Gonadal Sex Differentiation: Supporting Versus Steroidogenic Cell Lineage Specification in Mammals and Birds. Front. Cell Dev. Biol..

[65] Sengupta P., Dutta S., Karkada I.R., Chinni S.V. (2021). Endocrinopathies and Male Infertility. Life.

[66] Wu N., Mah C., Koentgen S., Zhang L., Grimm M.C., El-Omar E., Hold G.L. (2021). Inflammatory bowel disease and the gut microbiota. Proc. Nutr. Soc..

[67] Liao J., Liu Y., Yi J., Li Y., Li Q., Li Y., Shang P., Guo J., Hu L., Pan J. (2022). Gut microbiota disturbance exaggerates battery wastewater-induced hepatotoxicity through a gut-liver axis. Sci. Total Environ..

[68] Ferasyi T.R., Barrett P.H., Blache D., Martin G.B. (2016). Modeling the Male Reproductive Endocrine Axis: Potential Role for a Delay Mechanism in the Inhibitory Action of Gonadal Steroids on GnRH Pulse Frequency. Endocrinology.

[69] Kaprara A., Huhtaniemi I.T. (2018). The hypothalamus-pituitary-gonad axis: Tales of mice and men. Metabolism.

[70] Smith H.S., Elliott J.A. (2012). Opioid-induced androgen deficiency (OPIAD). Pain. Physician.

[71] Smith L.B., Walker W.H. (2014). The regulation of spermatogenesis by androgens. Semin. Cell Dev. Biol..

[72] Meccariello R., Chianese R., Chioccarelli T., Ciaramella V., Fasano S., Pierantoni R., Cobellis G. (2014). Intra-testicular signals regulate germ cell progression and production of qualitatively mature spermatozoa in vertebrates. Front. Endocrinol..

[73] Zhang H., Chang Y.F., Liu J. (2022). Editorial: Regulation of Mitochondrial Function on Animal Diseases. Front. Vet. Sci..

[74] Rato L., Alves M.G., Socorro S., Duarte A.I., Cavaco J.E., Oliveira P.F. (2012). Metabolic regulation is important for spermatogenesis. Nat. Rev. Urol..

[75] Rossi P., Dolci S. (2013). Paracrine mechanisms involved in the control of early stages of Mammalian spermatogenesis. Front. Endocrinol..

[76] Rato L., Meneses M.J., Silva B.M., Sousa M., Alves M.G., Oliveira P.F. (2016). New insights on hormones and factors that modulate Sertoli cell metabolism. Histol. Histopathol..

[77] Oliveira P.F., Alves M.G., Rato L., Silva J., Sa R., Barros A., Sousa M., Carvalho R.A., Cavaco J.E., Socorro S. (2011). Influence of 5alpha-dihydrotestosterone and 17beta-estradiol on human Sertoli cells metabolism. Int. J. Androl..

[78] Zirkin B.R., Papadopoulos V. (2018). Leydig cells: Formation, function, and regulation. Biol. Reprod..

[79] Weckman A., Di Ieva A., Rotondo F., Syro L.V., Ortiz L.D., Kovacs K., Cusimano M.D. (2014). Autophagy in the endocrine glands. J. Mol. Endocrinol..

[80] Organski A.C., Jorgensen J.S., Cross T.L. (2021). Involving the life inside: The complex interplay between reproductive axis hormones and gut microbiota. Curr. Opin. Endocr. Metab. Res..

[81] Ullah R., Naz R., Batool A., Wazir M., Rahman T.U., Nabi G., Wahab F., Fu J., Shahab M. (2021). RF9 Rescues Cortisol-Induced Repression of Testosterone Levels in Adult Male Macaques. Front. Physiol..

[82] Dehdari E.N., Sadeghi A., Ala M., Ebrahimi F., Pakbaz S., Azarpira N. (2023). Protective effects of melatonin against oxidative stress induced by metabolic disorders in the male reproductive system: A systematic review and meta-analysis of rodent models. Front. Endocrinol..

[83] Wynne K., Devereaux B., Dornhorst A. (2019). Diabetes of the exocrine pancreas. J. Gastroenterol. Hepatol..

[84] Craig J.R., Jenkins T.G., Carrell D.T., Hotaling J.M. (2017). Obesity, male infertility, and the sperm epigenome. Fertil. Steril..

[85] Osadchiy V., Martin C.R., Mayer E.A. (2019). The Gut-Brain Axis and the Microbiome: Mechanisms and Clinical Implications. Clin. Gastroenterol. Hepatol..

[86] Marsiglia R., Marangelo C., Vernocchi P., Scanu M., Pane S., Russo A., Guanziroli E., Del C.F., Valeriani M., Molteni F. (2023). Gut Microbiota Ecological and Functional Modulation in Post-Stroke Recovery Patients: An Italian Study. Microorganisms.

[87] Tolhurst G., Heffron H., Lam Y.S., Parker H.E., Habib A.M., Diakogiannaki E., Cameron J., Grosse J., Reimann F., Gribble F.M. (2012). Short-chain fatty acids stimulate glucagon-like peptide-1 secretion via the G-protein-coupled receptor FFAR2. Diabetes.

[88] Yano J.M., Yu K., Donaldson G.P., Shastri G.G., Ann P., Ma L., Nagler C.R., Ismagilov R.F., Mazmanian S.K., Hsiao E.Y. (2015). Indigenous bacteria from the gut microbiota regulate host serotonin biosynthesis. Cell.

[89] Marcelin G., Jo Y.H., Li X., Schwartz G.J., Zhang Y., Dun N.J., Lyu R.M., Blouet C., Chang J.K., Chua S.J. (2014). Central action of FGF19 reduces hypothalamic AGRP/NPY neuron activity and improves glucose metabolism. Mol. Metab..

[90] Perry R.J., Lee S., Ma L., Zhang D., Schlessinger J., Shulman G.I. (2015). FGF1 and FGF19 reverse diabetes by suppression of the hypothalamic-pituitary-adrenal axis. Nat. Commun..

[91] Xiong Y., Miyamoto N., Shibata K., Valasek M.A., Motoike T., Kedzierski R.M., Yanagisawa M. (2004). Short-chain fatty acids stimulate leptin production in adipocytes through the G protein-coupled receptor GPR41. Proc. Natl. Acad. Sci. USA.

[92] Palazzo M., Balsari A., Rossini A., Selleri S., Calcaterra C., Gariboldi S., Zanobbio L., Arnaboldi F., Shirai Y.F., Serrao G. (2007). Activation of enteroendocrine cells via TLRs induces hormone, chemokine, and defensin secretion. J. Immunol..

[93] Bravo J.A., Forsythe P., Chew M.V., Escaravage E., Savignac H.M., Dinan T.G., Bienenstock J., Cryan J.F. (2011). Ingestion of *Lactobacillus* strain regulates emotional behavior and central GABA receptor expression in a mouse via the vagus nerve. Proc. Natl. Acad. Sci. USA.

[94] Goehler L.E., Gaykema R.P., Opitz N., Reddaway R., Badr N., Lyte M. (2005). Activation in vagal afferents and central autonomic pathways: Early responses to intestinal infection with *Campylobacter jejuni*. Brain Behav. Immun..

[95] Cryan J.F., Dinan T.G. (2012). Mind-altering microorganisms: The impact of the gut microbiota on brain and behaviour. Nat. Rev. Neurosci..

[96] Sampson T.R., Mazmanian S.K. (2015). Control of brain development, function, and behavior by the microbiome. Cell Host Microbe.

[97] Nayak D., Roth T.L., McGavern D.B. (2014). Microglia development and function. Annu. Rev. Immunol..

[98] Nayak D., Zinselmeyer B.H., Corps K.N., McGavern D.B. (2012). In vivo dynamics of innate immune sentinels in the CNS. Intravital.

[99] Acevedo-Rodriguez A., Kauffman A.S., Cherrington B.D., Borges C.S., Roepke T.A., Laconi M. (2018). Emerging insights into hypothalamic-pituitary-gonadal axis regulation and interaction with stress signalling. J. Neuroendocr..

[100] Takahashi A., Kanda S., Abe T., Oka Y. (2016). Evolution of the Hypothalamic-Pituitary-Gonadal Axis Regulation in Vertebrates Revealed by Knockout Medaka. Endocrinology.

[101] May A., Leone M., Boecker H., Sprenger T., Juergens T., Bussone G., Tolle T.R. (2006). Hypothalamic deep brain stimulation in positron emission tomography. J. Neurosci..

[102] Sower S.A., Freamat M., Kavanaugh S.I. (2009). The origins of the vertebrate hypothalamic-pituitary-gonadal (HPG) and hypothalamic-pituitary-thyroid (HPT) endocrine systems: New insights from lampreys. Gen. Comp. Endocrinol..

[103] Nozaki M. (2013). Hypothalamic-pituitary-gonadal endocrine system in the hagfish. Front. Endocrinol..

[104] Zheng C.Y., Yu Y.X., Cao S.Y., Bai X. (2024). Epigenetics of inflammation in hypothalamus pituitary gonadal and neuroendocrine disorders. Semin. Cell Dev. Biol..

[105] Agarwal A., Saleh R.A., Bedaiwy M.A. (2003). Role of reactive oxygen species in the pathophysiology of human reproduction. Fertil. Steril..

[106] Barbagallo F., La Vignera S., Cannarella R., Aversa A., Calogero A.E., Condorelli R.A. (2020). Evaluation of Sperm Mitochondrial Function: A Key Organelle for Sperm Motility. J. Clin. Med..

[107] Weiss G.A., Hennet T. (2017). Mechanisms and consequences of intestinal dysbiosis. Cell Mol. Life Sci..

[108] Stojanov M., Baud D., Greub G., Vulliemoz N. (2018). Male infertility: The intracellular bacterial hypothesis. New Microbes New Infect..

[109] Sun Q., Wu S., Liu K., Li Y., Mehmood K., Nazar M., Hu L., Pan J., Tang Z., Liao J. (2023). miR-181b-1-3p affects the proliferation and differentiation of chondrocytes in TD broilers through the WIF1/Wnt/beta-catenin pathway. Pestic. Biochem. Physiol..

[110] Liu Y., Yi J., Li Y., Hussain R., Zhu S., Li Y., Ouyang Z., Mehmood K., Hu L., Pan J. (2022). Residue of thiram in food, suppresses immune system stress signals and disturbs sphingolipid metabolism in chickens. Vet. Immunol. Immunopathol..

[111] Zhu S., Liu Y., Li Y., Yi J., Yang B., Li Y., Ouyang Z., Liu B., Shang P., Mehmood K. (2022). The potential risks of herbicide butachlor to immunotoxicity via induction of autophagy and apoptosis in the spleen. Chemosphere.

[112] Wu X., Liu Y., Li Y., Tang Z., Li A., Zhang H. (2024). Molecular mechanism of thiram-induced abnormal chondrocyte proliferation via lncRNA MSTRG.74.1-BNIP3 axis. Pestic. Biochem. Phys..

[113] Wu S., Liu K., Huang X., Sun Q., Wu X., Mehmood K., Li Y., Zhang H. (2024). Molecular mechanism of miR-203a targeting Runx2 to regulate thiram induced-chondrocyte development. Pestic. Biochem. Phys..

[114] Sindi R.A., Alam S., Rizwan M., Ullah M.I., Ijaz N., Iqbal Z., Muzafar R., Akram R., Nazar M.W., Hussain R. (2023). Investigations of Hemato-Biochemical, Histopathological, Oxidative Stress and Reproductive Effects of Thiram in Albino Rats. Pak. Vet. J..

[115] Gyawali I., Zhou G., Xu G., Li G., Wang Y., Zeng Y., Li J., Zhou J., Zhu C., Shu G. (2023). Supplementation of microencapsulated probiotics modulates gut health and intestinal microbiota. Food Sci. Nutr..

[116] Galanis A. (2023). Shaping the Future of Probiotics: Novel Methodologies, Applications, and Mechanisms of Action. Microorganisms.

[117] Gul S.T., Alsayeqh A.F. (2022). Probiotics as an Alternative Approach to Antibiotics for Safe Poultry Meat Production. Pak. Vet. J..

[118] Hu S., Wang L., Jiang Z. (2017). Dietary Additive Probiotics Modulation of the Intestinal Microbiota. Protein Pept. Lett..

[119] Chibbar R., Dieleman L.A. (2015). Probiotics in the Management of Ulcerative Colitis. J. Clin. Gastroenterol..

[120] Ooijevaar R.E., Terveer E.M., Verspaget H.W., Kuijper E.J., Keller J.J. (2019). Clinical Application and Potential of Fecal Microbiota Transplantation. Annu. Rev. Med..

[121] Wang L., Gou X., Ding Y., Liu J., Wang Y., Wang Y., Zhang J., Du L., Peng W., Fan G. (2023). The interplay between herbal medicines and gut microbiota in metabolic diseases. Front. Pharmacol..

